# Preparation and Biocompatibility of Poly Methyl Methacrylate (PMMA)-Mesoporous Bioactive Glass (MBG) Composite Scaffolds

**DOI:** 10.3390/gels7040180

**Published:** 2021-10-23

**Authors:** Irina Atkinson, Ana Maria Seciu-Grama, Oana Catalina Mocioiu, Ana Maria Mocioiu, Luminita Predoana, Mariana Voicescu, Jeanina Pandele Cusu, Ramona Marina Grigorescu, Rodica Mariana Ion, Oana Craciunescu

**Affiliations:** 1 Romanian Academy, “Ilie Murgulescu” Institute of Physical Chemistry, 202, Spl. Independentei, 060021 Bucharest, Romania; omocioiu@icf.ro (O.C.M.); lpredoana@icf.ro (L.P.); voicescu@icf.ro (M.V.); jeaninamirea@yahoo.com (J.P.C.); 2 National Institute of Research and Development for Biological Sciences, 296, Spl. Independentei, 060031 Bucharest, Romania; oana_craciunescu2009@yahoo.com; 3 National R&D Institute for Non-ferrous and Rare Metals, 102, Biruintei Blvd, 077145 Pantelimon, Ilfov, Romania; ammocioiu@imnr.ro; 4 National Institute for Research & Development in Chemistry and Petrochemistry—ICECHIM Bucharest, 202, Spl. Independentei, 060021 Bucharest, Romania; ramona.grigorescu@icechim.ro (R.M.G.); rodica_ion2000@yahoo.co.uk (R.M.I.)

**Keywords:** biocompatibility, ceria, polymer-bioglass scaffolds

## Abstract

In recent years, the rising number of bone diseases which affect millions of people worldwide has led to an increased demand for materials with restoring and augmentation properties that can be used in therapies for bone pathologies. In this work, PMMA- MBG composite scaffolds containing ceria (0, 1, 3 mol%) were obtained by the phase separation method. The obtained composite scaffolds were characterized by X-ray diffraction, infrared spectroscopy, and scanning electron microscopy. UV–Vis measurement and EDX analysis confirmed the presence of cerium ions in the composite scaffolds. Evaluation of the in-vitro biocompatibility using MTT assay showed that composite scaffold containing 1 mol% of ceria presented higher viability than control cells (100%) for concentrations ranging between 5 and 50% after 96 h of incubation.

## 1. Introduction

Developments in tissue engineering have raised significantly the potential for treating bone defects caused by trauma, tissue resection, congenital anomalies, cancer, and osteoporosis [1]. Recently, scaffolds manufactured from natural or synthetic materials that provide structural support allowing cell proliferation upon transplantation, have become one of the important elements for regenerative medicine [2]. Moreover, the synthetic scaffolds can overcome limitations in current treatments associated with autologous bone grafting such as immunological rejection, the possibility of transmitting infectious diseases and low tissue availability [3,4]. Various types of scaffolds have been produced, but the main challenge facing us today is the selection of appropriate materials for scaffold manufacturing. To obtain scaffolds with the best properties, different types of materials have been used, such as natural or synthetic polymers, bioglasses, ceramics, metals, composites, and hydrogels. In addition, mechanical properties, biocompatibility, bioactivity, surface properties and biodegradability are essential in regenerative medicine applications and need to be considered when designing a scaffold [5].

Polymer/bioglass composite synthesis and development have played a key role in the advancement of biomedical technologies, including tissue engineering. The polymer/bioglass composite scaffolds combine two types of materials e.g., polymers and bio-glass to overcome their disadvantages and eventually produce a scaffold with superior properties [6].

Bioglasses used in synthetic scaffolds preparation for bone regeneration are attractive materials due to their ability to induce in-vitro hydroxyapatite mineralization and have excellent cytocompatibility [7]. Furthermore, bioglasses can be doped with different functional elements to enhance their biological properties [7,8].

To date, polymers have been the material of choice in the field of tissue engineering. PMMA acrylic bone cement has been widely used to repair or replace joints [9] and is used in a variety of medicinal and dentistry applications [10,11,12,13]. Polymers are extensively used in in-vivo and in-vitro biomedical applications due to their aesthetic, injecting molding ability [14]. Furthermore, PMMA is non-toxic, offers good compressive resistance and shows good versatile processing capabilities.

Several preparation techniques such as freeze-drying [15], phase separation [5], solvent casting [16], and matrix-assisted pulsed laser [17] methods have been used to produce scaffold-based polymer matrixes with adequate properties for bone tissue engineering. Among them, the phase separation method is an easy and simple way to obtain scaffolds that mimic bone morphology. Dhinasekaran D. et al. [5] obtained scaffolds by a phase separation method, with adequate properties of a bone grafting material starting from Bioglass 45S5 and PMMA using different solvents (ethanol, acetone, and chloroform). Studies regarding preparation PMMA-MBG scaffolds by phase separation method with the aid of nonionic surfactant Pluronic P123 are less frequently reported in the literature. Han X. et al. [18] obtained 3D Ti-doped meso-macroporous bioglass/PMMA scaffolds using Pluronic P123 and PMMA colloidal crystals by steam acid techniques. These scaffolds exhibit good antimicrobial properties and biocompatibility.

This study researches PMMA- Ce doped MBG composite scaffolds using the phase separation method. The influence of cerium addition on the biocompatibility of the obtained scaffolds using mouse fibroblast cells (NCTC clone L929) was investigated. In our previous study [8] good biocompatibility was obtained for Ce doped mesoporous bio-glasses based on 70SiO_2_–26CaO–4P_2_O_5_ system prepared by sol-gel method in the presence of surfactant Pluronic P123.

Among the therapeutic elements being more recently included in research studies is cerium. It has received particular interest due to its antibacterial, anti-inflammatory, pro-osteogenesis, and pro-angiogenesis properties due to the oxidation state transition Ce^4+^ and Ce^3+^ during redox reactions in physiological fluids with the formation of free radicals [19]. In addition, compressive strength and bioactivity of the obtained composite scaffolds were also studied.

## 2. Results and Discussion

### 2.1. FTIR

**Figure 1:**
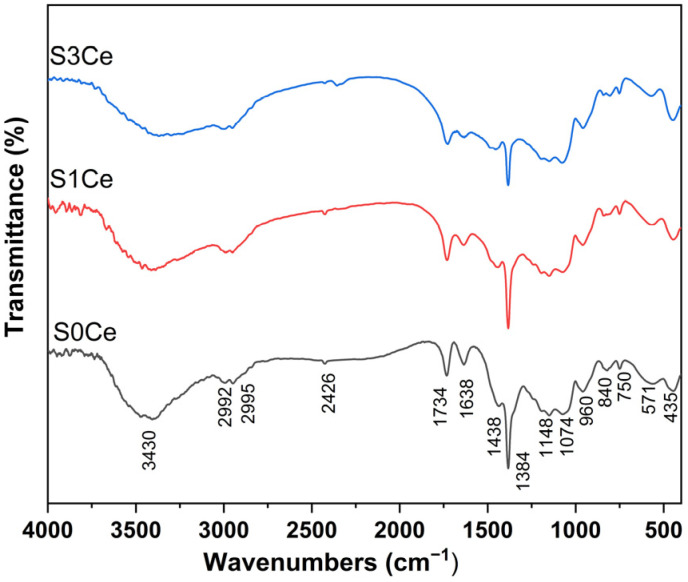
FTIR spectra of S0Ce-S3Ce composite scaffolds.

FTIR analysis was used to obtain information regarding chemical bonds. All of the prepared scaffolds (Figure 1) show the absorption bands at 2992, 2952 cm^−1^ assigned to C–H stretching vibrations related to the polymer matrix. The presence of hydrocarbon is indicated by the band at 1434 cm^−1^ due to the CH asymmetric bending vibration of CH_2_ [20]. Furthermore, the band at 1734 cm^−1^ correspond to the stretching vibration of the carbonyl (C=O) group while the band at 1638 cm^−1^ is due to C=C stretching vibration. The narrow and sharp band at 1384 cm^−1^ is attributed to the presence of NO^3−^ group. The absorption band at 3430 cm^−1^ corresponds to –OH stretching vibration (water or ethanol), while the band at 1638 cm^−1^ can be attributed to the interlayer stretching and bending vibration of molecular water [21].

The absorption bands located at 1074 cm^−1^ can be ascribed to Si–O–Si asymmetric stretching vibration and are due to the vibrations of the non-bridging Si–O bonds in the structural units Q3 which are tetrahedral [SiO4], with one non-bridging oxygen atom and three bridging oxygen atoms. It can be noticed that the band located at 1074 cm^−1^ slightly intensifies with the increase of cerium concentration, suggesting the depolymerization of Si–O network to units with less bridging oxygen and cerium acting as a network modifier in glass [22,23]. The bands situated at 750 and 435 cm^−1^ are due to Si–O–Si symmetric stretching of bridging oxygen and Si–O bending vibration, respectively. The presence of silanol is indicated with the band at 960 cm^−1^ due to Si-O stretching vibration on the silanol group [24]. The absorption bands at 850 and 570 cm^−1^ are related to the P–O vibrations.

The results indicate that in the bioglass solution under investigation, almost all of the alkoxy groups are hydrolyzed into silanol groups. According to [5], the addition of a hydrolyzed silica to the polymerized PMMA solution using ethanol and water as solvents can induce the phase separation which may be considered as (i) formation of glass network in the solution containing organic polymers; (ii) parallel growth of the bioglass network and the PMMA polymer; (iii) simultaneous growth of a bioglass–PMMA interconnected polymer network; (iv) and development of a bioglass–PMMA network connected by covalent bonds.

### 2.2. Thermal Analysis

**Figure 2:**
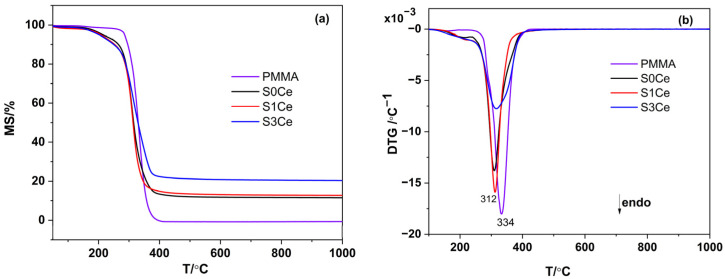
TG/DTA analyses of the pristine PMMA and PMMA-MBGs composite scaffolds: (**a**) TG analysis; (**b**) DTA analysis.

In order to examine the thermal stability, thermal analyses were carried out on PMMA-MBGs composite scaffolds as well as on pristine PMMA for comparison. The thermal gravimetric analysis (TG) and differential thermal analysis (DTA) data obtained from pristine PMMA and dried composite scaffolds are shown in Figure 2a,b. Both the pristine PMMA and the composite scaffolds underwent only single step degradation. The thermal decomposition for pure PMMA was completed around 400 °C. The onset of decomposition temperature decreased in the composite scaffolds, indicating the effect of cerium addition on the course of PMMA thermal degradation. No other effects were observed on the DTA curves.

### 2.3. UV-Vis

**Figure 3:**
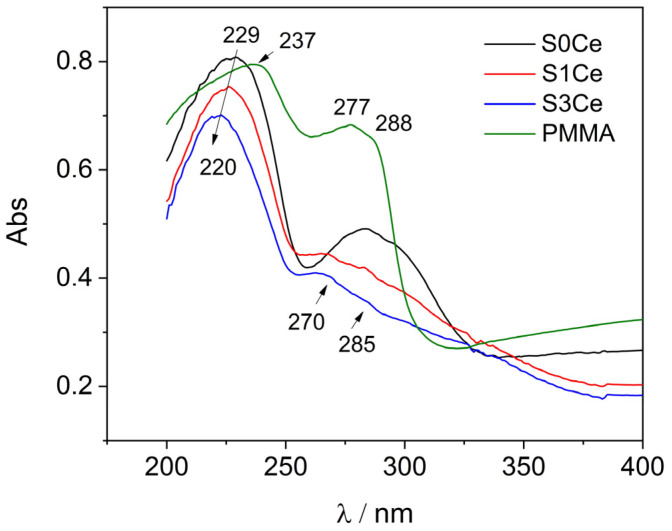
UV-Vis spectra of PMMA-MBGs composite scaffolds in direct comparison with pristine PMMA absorption spectrum.

UV-Vis analysis (Figure 3) was performed to obtain information regarding the oxidation state of cerium in the PMMA-MBGs composite scaffolds.

**Figure 4:**
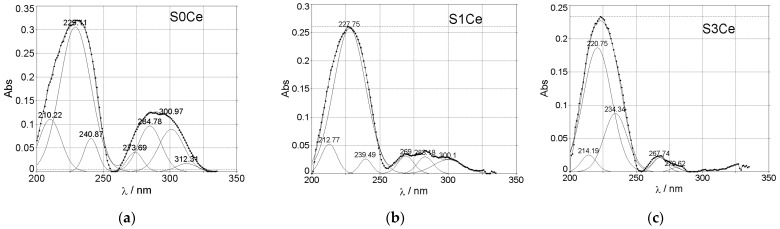
The curve fitting of the cerium (Ce^4+^/Ce^3+^) absorption band in PMMA-MBGs composite scaffolds: (**a**) S0Ce composite scaffold; (**b**) S1Ce composite scaffold; (**c**) S3Ce composite scaffold.

Likewise, spectra of the S0Ce and cerium doped composites scaffolds show an absorption band at about 229 nm attributed to PMMA. According to Aziz et al. [25] in the UV region, a sharp absorption edge of about 270 nm due to electronic transitions *n* ^®^ σ * occurs. Recently, Matamoros-Ambrocio et al. [26], observed that for PMMA microspheres in powdered form, synthesized under different conditions, all spectra in the UV region (200–400 nm) show a pronounced absorbance edge. On the other hand, in the Vis region (400–800 nm), the absorption is small and almost constant. This band is attenuated with the incorporation of cerium which can be associated with the band-gap absorption of CeO_2_ and hypochromic shifted with the absorption intensity linearly decreasing from S0Ce to S3Ce. It is known that significant changes in the absorption spectra of Ce^4+^ due to the composition of glass occur, the molar extinction coefficient of Ce^4+^ being 5 to 10 times stronger than that of Ce^3+^ [27]. Moreover, Ce^4+^ produces a very strong and broad charge transfer band around 250 nm, with the intensity, half-width, and position of the absorption wavelength changing significantly with glass composition [27]. Therefore, the hypochromic shift observed (S0Ce ^®^ S1Ce ^®^ S3Ce) which linearly decreases with the absorption intensity may be due to the incomplete inner electronic shell of Ce^4+^, as the scaffolds composite changed. Also, the charge transfer from O^2^^−^ to Ce^4+^ is taken into consideration. In the specific absorption spectra of Ce^3+^, within the 250–350 nm range, two well-structured absorption bands appear at about 270/285 nm [28]. According with Paul et al., Ce^3+^ in glass produces a number of absorption bands in the UV region corresponding to the f ^®^ d transitions [27]. Generally, Ce^4+^ is the stable valence in the cerium oxide due to its electronic configuration with the loss of a single electron in the 4f orbital of the Ce^3+^ ions [28]. The data from the literature reports the preparation of ceria-based oxides based on evaporation induces the self-assembly method using Pluronic P123 and ethanol as template removal, instead of the more common removal method (e.g., calcinations) [29]. Thus, the different type of spectrum pattern with the strong bleaching of the absorption band in the 270–280 nm region of S1Ce and S3Ce compared with S0Ce absorption band in this region, may be due to the fact that, by cerium doping of the composites’ scaffolds, a large accumulation of Ce^3+^ defects occurs. In this context, and based on the fact that cerium absorption overlaps with PMMA-MBG’s contribution, decomposition of the cerium (Ce^4+^/Ce^3+^) absorption band into the sample with Gaussian bands was performed (Figure 4). We conclude that, as composite scaffolds are doped with cerium, the concentration of Ce^4+^ increases while the concentration of Ce^3+^ decreases (Table 1). Moreover, the difference observed compared to that of pristine PMMA absorption may be due to porosity, defects, and oxygen vacancies in the PMMA structure of the scaffolds.

### 2.4. Morphology and Mechanical Properties Evaluation

**Figure 5:**
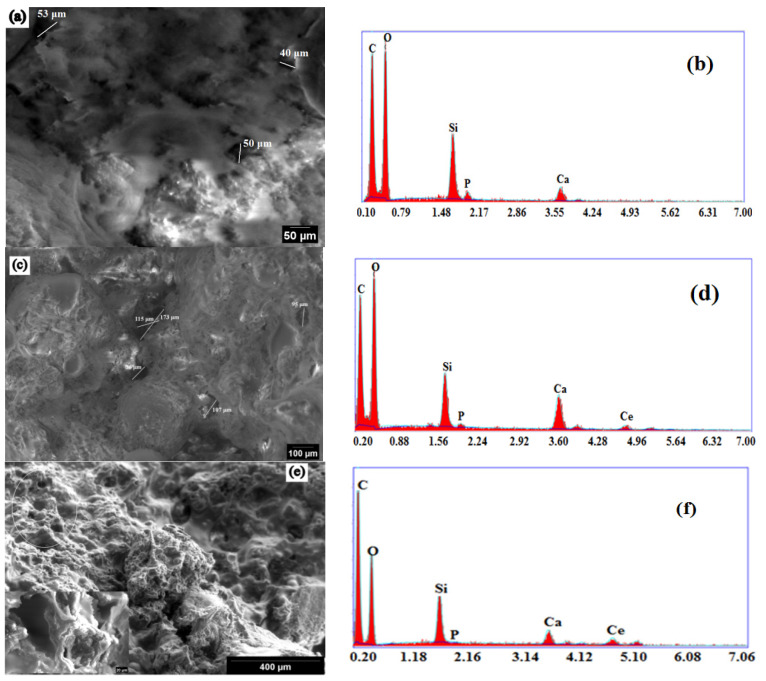
SEM micrographs (different magnifications) and EDX analysis of S0Ce (**a**,**b**), S1Ce(**c**,**d**), and S3Ce (**e**,**f**).

**Figure 6:**
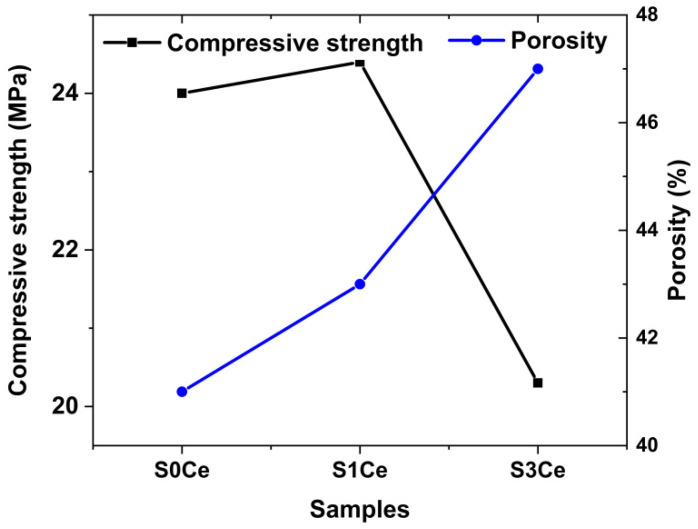
Porosity (blue line) and compressive strength (black line) of S0Ce-S1Ce composite scaffolds.

The morphology of the composite scaffolds surface and qualitative compositional analyses were evaluated by SEM. The SEM micrographs taken from the surface area of S0Ce-S3Ce samples are presented in Figure 5. Irregular micro-pores (less than 50 μm) were observed for S0Ce composite scaffold. It can be observed that the size and distribution of pores varies within different surface regions examined and increases in the cerium containing composite scaffolds. The size of the irregular pores was estimated to be in the range of 100 μm and 300 μm for S1Ce and S3 Ce composite scaffolds, respectively (Figure 5c,e). The formed pores can lead to some porosity which is one of the most important characteristics of the materials for tissue engineering applications. They also have an important role in bone regeneration and cell migration. The obtained composite scaffolds have a porosity between 41% and 47% (Figure 6). Depending on porosity, bone tissue can be classified in two categories: cortical bone (also known as dense bone) with a porosity of 5% to 15% and trabecular (cancellouse) bone with a porosity from 40% to 95% [30].

The results are in accordance with the data from the literature for materials with potential applications in tissue engineering (e.g., porosity in the range of 40–90%) [31]. In general, a pore size above 100 μm is required to promote the osteogenesis and angiogenesis [32]. Studies also report that a pore size under 100 μm plays a significant role in inducing the osteoinduction [33].

As can be observed in Figure 6, porosity and compressive strength are strongly correlated. The lowest compressive strength coefficient of 20 MPa is obtained for the S3Ce sample while the highest compressive strength value is found at 24.5 MPa for the S1Ce sample. The decrease of compressive strength coefficient with the increase of ceria content was reported by Zhou et al. [34]. An increase of the compressive strength of foamed glass-ceramics for a 2.5 wt% CeO_2_ addition was observed. Further increases of CeO_2_ content determine the decrease of the compressive strength. The higher content of Ce^4+^ as was revealed by UV-Vis analysis can lead to aggregation effect and consequently to distortion of the glass matrix. The data from the literature reported that the compressive strength of bioactive glass scaffolds ranges from 0.2 to 150 MPa depending on the scaffold’s composition, microstructure, and preparation method [35,36]. It is known that while the compressive strength of human cortical bone ranges between 90 and 209 MPa, the strength of cancellous bone is between 1.5 and 45 MPa [37]. Hence, the obtained values of compressive strength in the present study classify the scaffold as being a material that has promising mechanical behavior for application as a substitute of cancellous bone.

### 2.5. In Vitro Bioactivity Assessment

**Figure 7:**
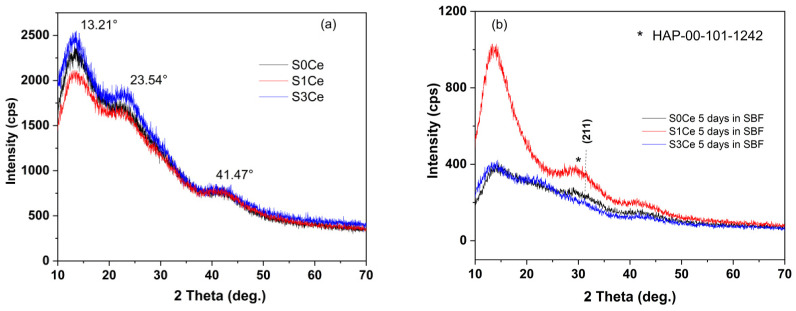
XRD patterns of S0Ce-S3Ce composite scaffolds (**a**) before immersion in SBF and (**b**) after immersion in SBF for five days.

The XRD patterns of PMMA-MBGs composite scaffold before and after immersion in SBF are depicted in Figure 7. The XRD patterns of the sample before immersion in SBF show broad diffraction lines of PMMA at 2θ 13.21° along with low intensity diffraction lines at 23.54° and 41.47° [38]. No diffraction lines corresponding to SiO_2_ or cerium oxides were observed.

After five days of immersion, XRD patterns of the S0Ce and the S1Ce samples reveal the beginning of hydroxyapatite (HAP) crystallization at 2θ around 31° (overlapping of (211), (112) and (300) reflection planes) in agreement with data from JCPDS card no. 101-1242. The overlapping of (211), (112) and (300) reflection planes is a characteristic of low crystalline HAP. Similar XRD patterns for PMMA-45S5 bioglass composites were obtained by [5] after 28 days of immersion in SBF.

**Figure 8:**
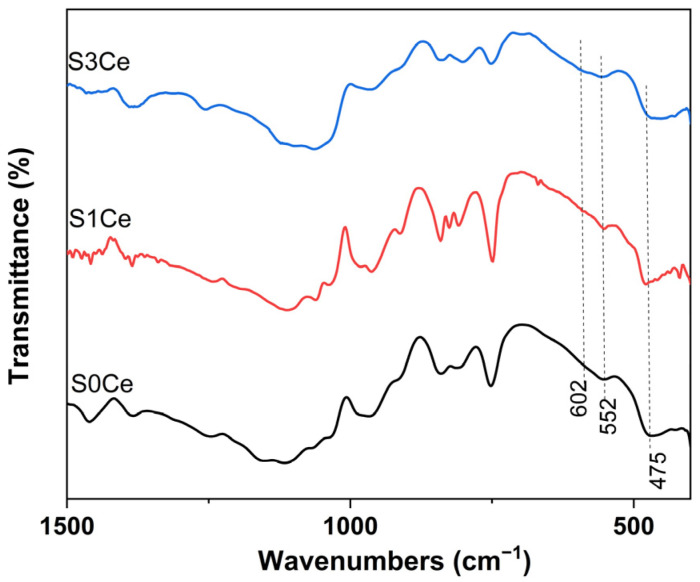
FTIR spectra of the PMMA-MBGs composite scaffolds immersed for five days in SBF solution.

Additionally, the formation of HAP layer on the surface of the composite scaffolds was also monitored by Fourier transforms infrared spectroscopic technique. Figure 8 shows the FTIR spectra of the obtained scaffolds immersed for five days in SBF solution. The characteristic absorption bands for phosphate group are observed at 552 and 602 cm^−1^ which are assigned to P–O bending vibration (ν4). The vibration band at 475 cm^−1^ corresponds to the PO_4_^−3^ bending vibration (ν2) [39]. The bands, assigned to P–O bending vibration modes of the orthophosphate PO_4_^−3^ group, are generally used to monitor the bioactivity [40].

**Figure 9:**
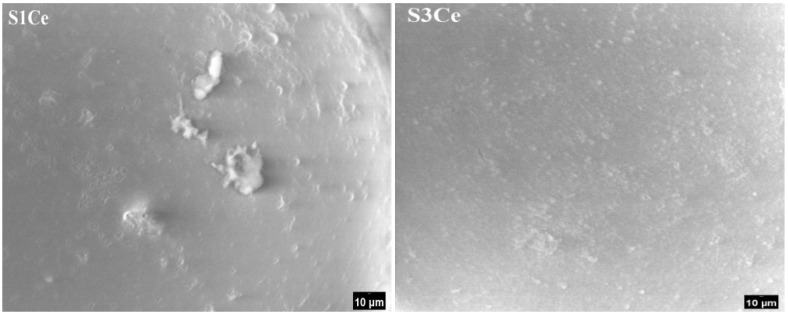
SEM micrographs of the S1Ce and S3Ce composite scaffolds after immersion for five days in SBF solution.

The surface of the S1Ce and S3Ce composite scaffolds after immersion in SBF for 5 five was also examined by SEM. As can be observed in Figure 9, the surface of samples is covered by fine particles confirming the beginning of HAP crystallization (as was revealed by XRD measurements). 

### 2.6. Biocompatibility Evaluation

**Figure 10:**
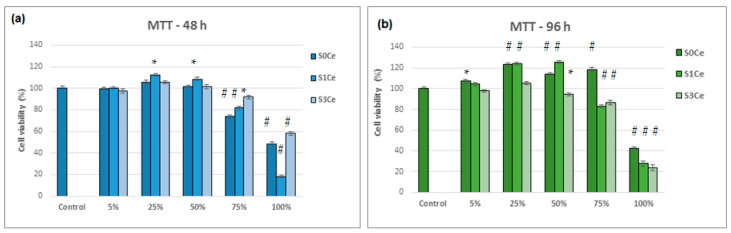
Viability of L929 cells exposed to different concentrations of PMMA-MBGs composite scaffolds evaluated by MTT assay after 48 h (**a**) and 96 h (**b**). Data are presented as mean ± SD (*n* = 3). * *p* < 0.05 compared to control (untreated cells); # *p* < 0.01 compared to control (untreated cells).

MTT assay (Figure 10) was used to evaluate indirect toxicity and the number of metabolic-active cells. Viability of L929 cells exposed to different concentrations of PMMA MBGs composite scaffolds was evaluated after 48 h (a) and 96 h (b). Data are presented as mean ± SD (*n* = 3). * *p* < 0.05 compared to control (untreated cells); # *p* < 0.05 compared to scaffolds-treated cells.

All tested samples show no cell cytotoxic activity in the concentration range between 5% and 75%, as seen in Figure 9. For all of the composite scaffolds produced during the investigation, the cell viability was above 80% (non-cytotoxic) for the aforementioned concentration range with exposure times of 96 h. At concentrations ranging between 5 and 50%, the S1Ce composite scaffold presented higher cell viability than control cells (100%) after 96 h of incubation. Good cell viability (84.73%) at a concentration of 100% was obtained for the MBGs containing 1% mole ceria in our previous study [8]. For the S0Ce composite scaffold, the cell viability was higher than control cells within concentrations ranging from 5 to 75% (Figure 10b). At 100% concentration, cell viability decreased drastically by up to 40% for the S0Ce after 96 h of incubation. The lowest cell viability after 96 h of incubation was observed for the S3Ce composite scaffold. This result can be explained based on the Ce^4+^/Ce^3+^ ratio. Naganuma et al. [41] reported that cell proliferation and adhesion in cerium-doped materials are influenced by the oxidation state of cerium (Ce^3+^ vs. Ce^4+^): Ce^3+^ ions inhibit cell proliferation and Ce^4+^ ions promote cell proliferation. In addition, the size and shape of CeO_2_ can influence its cytotoxicity with smaller sized CeO_2_ exhibiting higher toxicity [42].

## 3. Conclusions

PMMA-Ce doped MBG composite scaffolds with promising potential for application in tissue engineering were prepared by phase separation method by combining MBGs with addition of 0, 1, and 3 mol% ceria and PMMA.

UV-Vis measurements confirm both Ce^3+^ and Ce^4+^ oxidation states.

The compressive strength of the obtained composite scaffolds varies between 20–24.5 MPa that classify them as promising materials for application as a substitute of cancellous bone.

An in vitro biocompatibility evaluation determined using MTT assay indicated that all tested samples showed no cell cytotoxic activity on L929 cells in the concentration range of 5–75% after 96 h of incubation. Between concentration ranges of 5% and 50%, the S0Ce and S1Ce samples exhibited higher cell viability than control cells (100%).

XRD, FTIR, and SEM analyses confirmed the beginning of the hydroxyapatite layer crystallization over the sample surfaces after incubation in SBF for 5 days.

Based on the promising results, the PMMA-MBGs composite scaffolds investigated in the present study show potential for bone regeneration applications.

## 4. Materials and Methods

### 4.1. Reagents

This study used the following reagents: tetraethylorthosilicate (TEOS) (98%, Sigma-Aldrich, Darmstadt Germany), triethylphosphate (TEP) (99% Sigma-Aldrich, Darmstadt, Germany), calcium nitrate tetrahydrated (Ca(NO_3_)_2_·4H_2_O) (99% Sigma-Aldrich, Darmstadt, Germany) and cerium(III) nitrate hexahydrate (99% Sigma-Aldrich, Darmstadt, Germany) as silica, phosphate-, calcium- and cerium-oxide precursors, respectively, hydrochloric acid (HCl) (Sigma-Aldrich, Darmstadt, Germany) as a catalyst, PEG-PPG-PEG, called Pluronic^®^ P123 (Sigma-Aldrich, Darmstadt, Germany) as structure directing agent and poly methyl methacrylate (Alfa Aesar, Ward Hill, MA, USA).

### 4.2. Preparation of MBG Solution

The bio-glass precursor sol was directly used to obtain the scaffolds. In brief, Ce-doped mesoporous bioglasses in the 70SiO_2_-(26-x) CaO-4P_2_O_5_-xCeO_2_ system (where x stands for 0, 1, 3 mol%) were synthesized using the procedure described in paper [8]. Pluronic P123 was used as a structure directing agent.

### 4.3. Preparation of the Polymer-MBG Scaffolds

**Figure 11:**
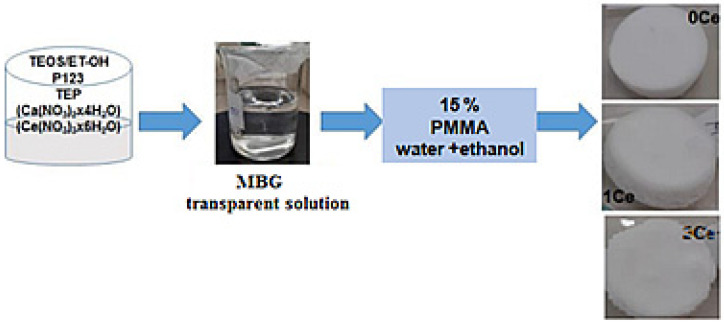
Composite scaffolds preparation chart.

PMMA-MBG scaffolds were prepared by the phase separation method following the procedure described in [5]. PMMA (15%) with a molecular weight of 550,000 and a density of 1.18 g cm^3^ was dissolved in an ethanol and water mix. Equal volumes of the MBG solution and the polymer/water/ethanol mixture were mixed to obtain the scaffold materials. Ethanol and water were mixed in the ratio 4:1 and preheated to 60 °C before adding PMMA. Subsequently, the obtained scaffolds were washed with ethanol to remove the Pluronic P123 structure directing agent and dried in the oven at 60 °C. The obtained scaffolds were labeled as follows: S0Ce, S1Ce, and S3Ce. The preparation chart is presented in Figure 11.

### 4.4. Composite Scaffolds Characterization

Powdered X-ray patterns of the scaffolds were recorded using a RigakuUltima IV diffractometer in parallel beam geometry equipped with CuKα radiation (wavelength 1.5406 Å) in 2θ range between 10 to 70 with a speed of 2°/min and a step size of 0.02°. PDXL software (Version 1.8) from Rigaku, connected to ICDD database was used for phase identification.

Fourier transform infrared (FTIR) spectroscopy was performed with a Nicolet Spectrometer 6700 FTIR, within 400–4000 cm^−1^ range, in transmittance mode.

The thermal behavior of the obtained MBG-composite scffolds was determined by thermal gravimetric analysis and differential thermal analysis (TG/DTA) using a Mettler TOLEDO TGA/SDTA 851e equipment in flowing air atmosphere using alumina crucible. The maximum temperature was set at 1000 °C and the heating rate was 10 °C/min

The absorption measurements were recorded with a Perkin Elmer Lambda 35 Spectrometer with integrating sphere in 900–200 nm range using: data interval, 1 nm; scan speed, 60 nm/min; slit, 4 nm; sample holder at 8° wedge and a certified reflectance standard. To estimate the cerium concentration into scaffolds, a least-squares iterative curve fitting was performed with Gaussian bands using the peak fit analysis program (Sea-Solve, Framingham, MA, USA). The areas of all bands assigned to a given concentration were summed up and divided by the total area in order to obtain the contribution of cerium (Ce^4+^/Ce^3+^).

The morphology of the composite scaffolds was investigated by scanning electron microscopy using a microscope; Quanta FEI 200 model coupled with energy dispersive X-ray (EDX) analysis.

The compressive strength of the composite scaffolds was evaluated using a portable Schmidt hammer. The rebound number can be converted to uniaxial compressive strength (UCS-MPa) that, according to Wang R. and Yuan Z. [43] can be correlated to a qualitative evaluation of sample hardness.

The porosity of the samples was determined using Archimedes method. The porosity was calculated using Equation (1).

(1) $$\mathrm{Porosity},\;\%=\frac{\left(m_{1}-m_{0}\right)}{\delta V_{0}}\times 100$$

where: m_0_ and m_1_ indicate the weight of the scaffold before and after immersing, respectively, V_0_ is the scaffold volume before immersing, and δ is the liquid density. The experiment was performed in triplicate.

In vitro bioactivity of the composite scaffolds was assessed by immersing the samples in the simulated body fluid (SBF) as proposed by Kokubo et al. [44] at 37 °C for five days Once removed from the incubation solution, the samples were washed with deionized water and dried at 70 °C for 24 h. The presence of newly formed hydroxyapatite on the surface of composites was further examined by XRD analysis.

In vitro evaluation of cell viability was performed using the MTT assay protocol, as described in [45]. To summarize, after both 48 h and 96 h of cultivation in the presence of the scaffolds, the cells were rinsed with phosphate buffered saline solution (PBS), pH 7.4, and incubated with an MTT working solution (0.25 mg/mL) for 3 h at 37 °C to obtain crystallized formazan. Afterwards, the medium was removed and isopropyl alcohol was added to each well. After incubation at room temperature for 15 min under gently stirring, the optical density of the solution was determined at 570 nm using a Tecan Sunrise microplate reader (Tecan, Austria). The amount of converted dye directly correlates to the number of metabolically active cells. Cell viability was expressed as a percentage of the control cells (cells incubated without sample) considered being as 100% viable. The tests were performed in triplicate.

## Figures and Tables

**Table 1:** Percentage of Ce^4+^/Ce^3+^ in PMMA-MBGs composite scaffolds calculated from the fitting of Gaussians in the UV region, with the corresponding positions indicated in parentheses.

Sample	PMMA-MBGs	Ce^4+^	Ce^3+^
S0Ce	13.40% (210.22 nm)	-	-
	51.70% (229.11 nm)	-	-
	5.29% (240.87 nm)	-	-
	3.42% (273.69 nm)	-	-
	12.10% (284.78 nm)	-	-
	12.18% (300.97 nm)	-	-
	1.91% (312.31 nm)	-	-
S1Ce	-	7.72% (212.77 nm)	4.59% (269.00 nm)
	72.26% (227.75 nm)	-	4.34% (283.48 nm)
	7.43% (300.10 nm)	3.66% (239.49 nm)	-
S3Ce	-	5.26% (214.19 nm)	3.80% (267.74 nm)
	65.32% (220.75 nm)	-	1.00% (279.62 nm)
	-	24.62% (234.34 nm)	-

## Data Availability

The data presented in this study are contained within the article.
